# Supplementary material for: Patient Perspectives on Coordinated Care: Preliminary Results from the Implementation Stage Using Patient-Reported Experience Measures (PREMs)
Source: Healthcare (Basel). 2025 Apr 29;13(9):1026. doi: 10.3390/healthcare13091026 (PMC12071257; doi:10.3390/healthcare13091026)
Supplement: Supplementary file 1 [file healthcare-13-01026-s001.zip › healthcare-3535369-supplementary.pdf]

Lp.	Statements	Rating 1-5				
		Never (1)	Rarely (2)	Sometimes (3)	Almost always (4)	Always (5)
A. Patient's active participation in the treatment process						
1 a.	My doctor appreciates my opinion about the treatment, its course and results.					
2 a.	My doctor has enough time to listen to me and answer my questions.					
3 a.	My doctor discusses with me the next steps in the treatment of my disease.					
4 a.	My doctor gives me the opportunity to choose the method of treatment, if there are different methods of therapy (treatment) for my disease.					
5 a.	My doctor wants me to talk about various issues related to my health, such as side effects of medications used, the course of other conditions.					
B. Patient support in decision making						
6 b.	My doctor informs me of additional tests and medical consultations recommended for my chronic disease.					
7 b.	My doctor or another member of the medical staff helps me determine what needs to be changed in my lifestyle to better manage my chronic disease.					
8b.	My doctor or another member of the medical staff gives me information materials (brochures, leaflets) explaining how to deal with my disease.					
9 b.	I have free access to my medical records.					
C. Continuity of care						
C1. Relational continuity						
10 c.	I have a doctor assigned.					
11 c.	I can continue specialized treatment without having to constantly visit the family doctor to get referrals.					
C2. Information continuity						
12 c.	My doctor has information about the recommendations that other doctors give me, what medications they prescribe for me, what procedures I have had in other medical institutions					
13c.	I have regular diagnostic tests and follow-up visits due to my chronic disease.					

D. Coordination of care						
14d.	I have a designated person from the medical staff (doctor, nurse) who organizes all my care in the health care system.					
15d.	The doctors who treat me do not contradict each other; I get consistent/the same medical advice from different doctors.					
16d.	My doctor consults other doctors to discuss my condition.					
17d.	I receive comprehensive healthcare related to my chronic disease - I can attend many different medical consultations in the same medical facility (e.g. with a dietitian, a physical therapist).					
E. Solving patient's problems						
E1. Solving patient's health and organizational problems						
18e.	My doctor explains the aims of the treatment, the treatment process and the expected results.					
19e.	My doctor or another member of the clinic staff explains to me what to do next with the treatment, where to go next, how to proceed, who to contact, etc.					
20e.	My doctor or another person from the medical staff gives me instructions on how to act in the event of a sudden deterioration in health, exacerbation of the signs and symptoms of the disease.					
E2. Building a sense of patient safety						
21e.	I can request a home visit from a doctor or nurse.					
22e.	I can contact the person responsible for my care (community nurse, my doctor) by phone or email.					
F. Flexibility and availability						
23f.	I can choose a convenient date for my visit.					
24f.	I have access to information about the services offered and the waiting time (e.g. by phone, on the website of the institution).					
25f.	The results of my laboratory tests are available on time, i.e. before my next visit at my doctor's.					

G. Patient-centredness						
26g.	The staff of the facility responds quickly to my needs; I feel the staff is interested in me.					
27g.	In the facility, there is access to information about the patient's rights, including the possibilities and ways of managing a complaint or contact with a supervisor (e.g. clinic manager).					
28g.	The facility where I am treated is interested in what patients think about it; it conducts satisfaction research.					
29g.	The facility where I am being treated is making changes that I believe will improve patient services.					
